# Risk Factors for Persistent Anosmia and Dysgeusia in Children with SARS-CoV-2 Infection: A Retrospective Study

**DOI:** 10.3390/children10030597

**Published:** 2023-03-21

**Authors:** Francesco Mariani, Rosa Morello, Daniele Omar Traini, Anna La Rocca, Cristina De Rose, Piero Valentini, Danilo Buonsenso

**Affiliations:** 1 Department of Woman and Child Health and Public Health, Fondazione Policlinico Universitario Agostino Gemelli IRCCS, 00168 Rome, Italy; 2 Medicine and Surgery, Catholic University of Rome, 00168 Rome, Italy; 3 Centro di Salute Globale, Università Cattolica del Sacro Cuore, 00168 Rome, Italy

**Keywords:** SARS-CoV-2, Long COVID, post-COVID-19 condition, anosmia, dysgeusia

## Abstract

Background: Olfactory and gustative dysfunctions are two of the most common post-acute sequelae of SARS-CoV-2 infection in children, which can have a negative impact on the routines of children and families. As several children have had COVID-19 since the Omicron variant, it is important to investigate if this increase in infections is reflected in higher olfactory/taste disfunctions. The primary aim of this study was to characterize the presence of olfactory/gustative problems in a cohort of children, its evolution, and its association with risk factors such as COVID-19 variant, hospitalization, presence of olfactory/gustative dysfunction during the acute phase, and vaccination. Methods: This was a retrospective analysis of children with microbiologically confirmed SARS-CoV-2 infection evaluated in person at a referral pediatric post-COVID-19 clinic in Rome, Italy. We included children younger than 19 years old, evaluated from the beginning of the pandemic up to October 2022. At specific timepoints, we investigated the presence of olfactory/taste disfunctions and evaluated them according to the SARS-CoV-2 variants circulating at the time of infection. Results: A total of 1250 children (650 females; 52.0%) with a mean age of 6.77 (±4.12) years were included in the study. At 3, 6, 12, and 18 months, 12 (9.6%), 7 (5.6%), 2 (1.6%), and 1 (0.8%) of the children reported anosmia and dysgeusia post-COVID-19 infection, respectively. The presence of anosmia and dysgeusia during the acute phase of infection and being infected with a pre-Omicron variant were found to be significant risk factors for persistent olfactory and gustatory dysfunction during all follow-up periods. Conclusions: anosmia and dysgeusia symptoms tended to decrease gradually over time, but not all children recovered quickly.

## 1. Introduction

Post-COVID-19 condition (PCC), Long COVID, and post-acute sequelae of SARS-CoV-2 infection (PASC) are the three most used denominations for expressing the combination of unexplained, persistent, residual, or new-onset symptoms after an encounter with COVID-19 SARS-CoV-2 infection [1]. The first pediatric definition of PPC was recently proposed in March 2022, after being developed through a Delphi process involving both researchers and family advocates. According to this definition, Long COVID in pediatric patients is described as “a condition that occurs in young people with a history of confirmed SARS-CoV-2 infection, with at least one persisting physical symptom for a minimum duration of 12 weeks after initial testing that cannot be explained by an alternative diagnosis. The symptoms have an impact on everyday functioning, may continue or develop after COVID-19 infection, and may fluctuate or relapse over time” [2]. This definition is in line with a newer one provided by the WHO specifically for children [3]. The physical symptoms, to which the definition refers, subsist mainly of chronic fatigue and neuropsychiatric, cardiovascular, pulmonary, hematologic, gastrointestinal, renal, endocrine, dermatologic, and musculoskeletal sequelae. Currently, there is increasing evidence that persistent olfactory dysfunction (anosmia) and taste dysfunction (dysgeusia) are frequent complications of acute SARS-CoV-2 infection in children [4]. Despite the growing impact of the latter two dysfunctions, not much is yet known about the pathophysiology underlying them. Distinctively, what is evident is that these conditions are capable of generating imperative modifications in the ordinary life of children. As a matter of fact, a reduced appetite and food enjoyment lead to a decreased nutritional intake, which in turn can induce depression and, ultimately, may precipitate in social isolation [5]. Due to these dramatic sequelae, it is imperative for pediatric researchers to focus further attention on the possible ways of preventing their occurrence. Accordingly, our study aimed to identify the events piloting the development of these conditions, together with the ones contrarily functioning as protective factors. In such a way, our intention was to become aware of the risk factors underlying persistent anosmia and dysgeusia, so that their prevention could be more accessible in the near future. An additional aspect to which our retrospective study gave thought was that although anosmia and dysgeusia have been largely described during the first waves of the pandemic [6,7], little is known about their presence and long-term persistence in children affected by the newer variants and according to their vaccination status. This lack of information represents a gap in the evidence that clinicians can use to discuss with parents and children about the risks of new infections and possible benefits from vaccination. In order to acquire such knowledge, we aimed to investigate the long-term duration of persisting anosmia and dysgeusia (at months T3, T6, and T12) in a large cohort of children with post-COVID-19 condition. Additionally, we aimed to examine the potential impact of the variants (original, Alpha, Delta, and Omicron) and the number of vaccination doses on the obtained outcomes.

## 2. Materials and Methods

### 2.1. Study Population and Setting

This was a retrospective analysis of children with microbiologically confirmed SARS-CoV-2 infection evaluated in person at a referral pediatric post-COVID-19 clinic in Rome, Italy (Ethic Approval ID 4518; Prot 0040139/21). We included children younger than 19 years of age referred to our outpatient clinic after a documented SARS-CoV-2 infection from the beginning of the pandemic up to October 2022. The study population included patients who were referred to our post-COVID-19 outpatient unit from other clinics. The exclusion criteria included patients older than 18 years, individuals with suspected but not lab-confirmed infection, individuals with ongoing acute infections, and children unable to refer to anosmia/dysgeusia.

For each patient, we collected information about the acute infection and persistence of anosmia/dysgeusia at 3, 6, 12, and 18 months after an acute SARS-CoV-2 infection. Data on demographics (age, gender, and pre-existing conditions) were collected, together with data on the severity of the acute infection (as previously defined in [8]), the outcome of the acute SARS-CoV-2 infection, and COVID-19 vaccination status (mRNA vaccines licensed in Italy). Data on the dominant circulating variant at the time of infection were collected from the report coordinated by the Italian Superior Health Institute [9].

### 2.2. Study Aims

The aim of this study was to evaluate the burden of anosmia and dysgeusia during acute infection and long-term follow-up in children infected with SARS-CoV-2. The secondary aim was to evaluate the impact of the SARS-CoV-2 variants and vaccination status on the persistence of anosmia/dysgeusia.

Concurrently, we contemplated the burden underlying the possible pathologies and comorbidities of children in the study, considering also whether the patient had ever been hospitalized or taken to an intensive care unit during their SARS-CoV-2 infection.

### 2.3. Statistical Analysis

Categorical variables were reported as a count and percentage. Continuous variables were expressed as a mean with standard deviation. The statistical association between the categorical variables was obtained by chi-squared tests or Fisher’s exact tests. The statistical analysis was performed using IBM SPSS Statistics 26.0 software (IBM Corporation, Armonk, NY, USA).

## 3. Results

**Figure 1:**
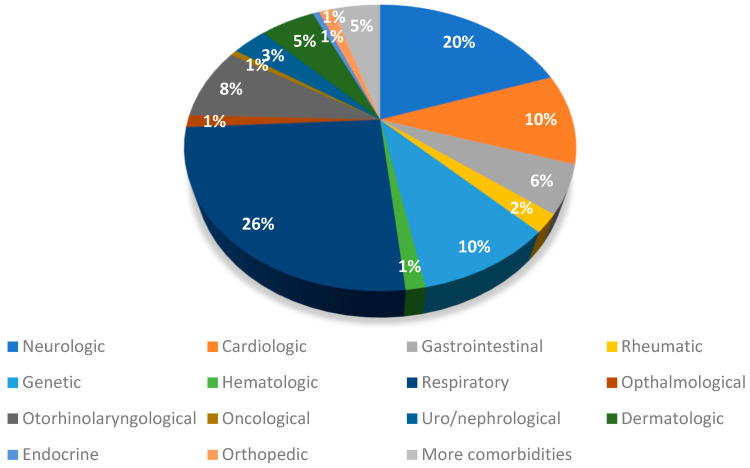
Comorbidities of the children in our cohort study.

We retrospectively reviewed the medical charts of 1250 children (52.0% females). The mean age was 6.77 (+/− 4.12) years. Among the total cohort, 144 individuals (11.5%) had pre-existing comorbidities, as outlined in Table 1. With regard to the severity of the acute phase of infection, 108 (8.6%) were asymptomatic, 1119 (89.5%) had a mild acute infection, and 23 (1.8%) had a moderate SARS-CoV-2 acute infection. The probable infectious variants were also assessed, with 37 children (3.0%) being affected by the original viral infection, 73 (5.8%) by the Alpha variant, 246 (19.7%) by the Delta variant, and 894 (71.5%) by the Omicron variant. Concerning the vaccinations, 1002 children (80.2%) were not vaccinated, whilst 248 (19.8%) had received at least 1 dose. The percentage of children who had received a complete vaccination with three doses was 13.4%. Comorbidities are listed in Figure 1.

Regarding the characteristics of the acute phase of the infection, 26 children (2.1%) were hospitalized and only 1 child (0.1%) needed a pediatric intensive care unit (PICU). A total of 64 patients (5.1%) presented anosmia during the acute phase and 53 children (4.2%) suffered from dysgeusia.

All the children (1250) underwent a follow-up at our pediatric clinic at 3 months (T3); 12 children (1.0%) presented anosmia and the exact same number of patients also presented dysgeusia. At 6 months (T6), 1224 (97.9%) children from the total cohort were assessed; 7 (0.6%) presented symptoms of anosmia and once more the equivalent cipher presented dysgeusia. At 12 months (T12), 181 patients (14.5%) were subjected to a follow-up; 3 of them (1.7%) had anosmia, whilst 2 (1.1%) had dysgeusia. Ultimately, 87 children (7.0%) were visited at 18 months (T18); only 2 (2.3%) were disturbed by anosmia and only 1 (1.1%) by dysgeusia. Those characteristics are reported in Table 2.

A comparison between children with anosmia and dysgeusia and children without those symptoms at the different follow-up evaluations is reported in Table 3, Table 4, Table 5 and Table 6.

At the three month follow-up, a statistically significative association was observed between the wild variant (*p* = 0.047), the Delta variant (*p* = 0.017), the Omicron variant (*p* = 0.001), acute anosmia (*p* < 0.001), and acute dysgeusia (*p* < 0.001) with anosmia (Table 3); the same association, except for the Delta variant, was statistically significative with the persistence of dysgeusia (Table 3).

At the six month evaluation, a statistically significative association was observed between the Omicron variant (*p* < 0.001), the Delta variant (*p* = 0.005), acute anosmia (*p* < 0.001), and acute dysgeusia (*p* < 0.001) with anosmia and dysgeusia persistence (Table 5 and Table 6).

At the 12 month follow-up, a statistically significative association was observed between acute anosmia (*p* = 0.003) and acute dysgeusia (*p* = 0.036) with anosmia (Table 7 and Table 8); regarding dysgeusia persistence, only dysgeusia during the acute phase was associated (*p* = 0.029) (Table 7 and Table 8).

At the 18 month evaluation, a statistically significative association was observed between acute anosmia (*p* = 0.028) and acute dysgeusia (*p* = 0.021) with anosmia and dysgeusia persistence (Table 9 and Table 10).

We also evaluated the proportion of vaccinated and fully vaccinated children (having received at least two doses) among those who developed these symptoms and those who did not. Our findings showed no association between the vaccination status and the persistence of dysgeusia or anosmia at different follow-up times.

## 4. Discussion

In this study, we found that a subgroup of children developed long-term olfactory/taste dysfunctions. Although most children recovered over time, a few children still complained about this symptom up to 18 months after infection. Importantly, children infected with the Omicron variant had a significantly lower risk of developing long-term dysfunction.

For over a year, the pediatric ward of Gemelli Hospital observed an increasing number of parents seeking care for their children, affected by persistent symptoms following SARS-CoV-2 infection. One of the most common sequelae observed over this time period was the persistence of anosmia together with the persistence of dysgeusia, in line with other studies [10,11,12,13,14,15,16,17,18,19,20,21,22,23,24,25,26,27,28,29,30,31,32,33,34]. Often, symptoms are so severe that they prevent children from eating normally, as they used to do pre-COVID-19. As it is possible to see from our study, the majority of children will recover taste and smell within 6 months; however, a minority (in our cohort, 2.3% for anosmia and 1.1% for dysgeusia) may remain affected by persistent symptoms of anosmia and dysgeusia for a timeframe of over 18 months. Our work was based on a broad child population and involved well-defined times of follow-up at 3, 6, 12, and 18 months after an encounter with acute SARS-CoV-2 infection. As such, we have provided the longest follow-up analysis of persisting symptoms after initial infections of children.

Through our study, we have provided—for the first time, to our knowledge—information about the impact of the new SARS-CoV-2 virus variants Alpha, Delta, and Omicron on loss of smell and loss of taste in children. It emerged that the Omicron variant was less likely to cause chronic anosmia and dysgeusia in children compared with the other variants. This is important information that can help clinicians in the communication with parents and patients about the risk of developing this complication and the usual time of recovery in newly infected children, which has not been described in recent major international cohorts [35]. From what was scientifically proven, the Omicron variant is distinguished by its steady capacity to spread more easily [36]. Curiously, this depends on its set of mutations; there are 15 mutations in the receptor-binding domain (the part of the spike that mediates the binding of the virus to the cell) and there are also 3 mutations near the furin cleavage site that may be involved in making the variant more transmissible. By the same token, a group of four new mutations could create additional obstacles for antibodies [37,38]. Despite it being such a feared variant, strictly because of the characteristics belonging to it, our study showed that it was associated in a reductive way with the emergence of anosmia and dysgeusia. In reverse, the original COVID-19 strain, together with the Delta variant, were more frequently associated with these symptoms.

Another key aspect is the role of vaccinations in PCC prevention. Our study identified a small, non-statistically significant protective effect of partial and full vaccination on preventing PCC after a breakthrough infection in children. The protective effects were more pronounced in children who had received two doses of the vaccine. This represents new information that clinicians can use to discuss and communicate with parents about other possible advantages of being vaccinated. Unfortunately, the number of children receiving three doses was too small to be included in separate analyses. There is only one other study that has addressed this issue in children. Messiah et al. showed that patients who did not report vaccination information were six times more likely to develop PCC than those who were vaccinated (RR: 5.76; 95% CI: 1.18–28.06) [39]. Studies in adults have shown a stronger protective effect of vaccinations, suggesting that vaccination before SARS-CoV-2 infection could reduce, but not eliminate, the risk of PCC. A literature review and meta-analysis including 18 studies, mostly from the USA, UK, and Spain, showed that the vaccinated group had a lower risk of developing persistent symptoms after SARS-CoV-2 infection compared with the unvaccinated group [40]. However, the protective effect was restricted to cognitive symptoms, kidney diseases, myalgia, and sleeping disorders. Similar findings have been reported in other studies in adults [40]. There have been several hypothetical physiologic mechanisms proposed to explain the protective effect, including a less severe illness with less organ damage following vaccination and a faster elimination of viral particles, reducing the risk of chronic inflammation [40,41]. A possible explanation for the lack of a full protective effect of vaccines identified in our study may be due to the low number who were fully vaccinated with three doses. Another hypothesis could be that protection provided by the vaccines wanes after 4 to 6 months [42]. Thus, moving away from vaccination, its possible protective effect may gradually decrease until it returns to the risk of the pre-vaccination status.

Our study has limitations to address as it was a retrospective study and smell/taste problems were defined according to a self-description of smell impairments by the patients in the absence of a standardized diagnostic tool. In addition, the infecting variants were not defined by genetic studies but according to the prevalent circulating variant in Italy at the time of infection.

## 5. Conclusions

Our study demonstrated the utmost imprint that Long COVID cedes on the pediatric population. With our heightened understanding of the pathological mechanisms concerning Long COVID, it is mandatory that long-term sequelae in children are not dismissed in the near and long future. A remarkable aspect that was concluded was that among the different SARS-CoV-2 variants, the Omicron variant was less likely a cause of chronic anosmia and dysgeusia in children. Indeed, our study observed that patients with these symptoms were significantly underrepresented among those infected with the Omicron variant, whereas the original COVID-19 strain and the Delta variant were more frequently associated with chronic neurosensory loss. Regarding the role of vaccination, our data did not demonstrate a strong correlation between vaccination and chronic post-COVID-19 olfactory/gustatory loss, although fewer children with at least two doses of vaccination developed persistent anosmia/dysgeusia. This suggested that vaccination may not play a significant partial protective role in protecting infected children from the long-term effects of COVID-19 on taste or smell. Additional studies are needed to understand if a three-dose course is more protective. However, it is important to consider additional data to reach a more definitive conclusion. Further studies are needed to fully understand the relationship between vaccination and these symptoms and to determine the best course of action to protect children from chronic anosmia and dysgeusia or to treat them once children develop these symptoms. For the time being, our research provides updated knowledge and guidance to families and to all the scientific society about SARS-CoV-2 infection residues in the pediatric population.

## Figures and Tables

**Table 1:** Clinical and demographic data of the study population.

	Total Cohort (N = 1250)
Age (years), median (IQR)	6.77 (+/−4.12)
Female, n (%)	650 (52.0%)
Comorbidities, n (%)	144 (11.5%)
Vaccination, n (%) *	248 (19.8%)
Acute phase severity, n (%) - Asymptomatic - Mild - Moderate	108 (8.6%) 1119 (89.5%) 23 (1.8%)
COVID-19 variant, n (%) - Original - Alpha - Omicron - Delta	37 (3.0%) 73 (5.8%) 894 (71.5%) 246 (19.7%)
	**Total cohort (N = 1245)**
* Complete vaccination, n (%)	167 (13.4%)

**Table 2:** Anosmia and dysgeusia of the study population at FUP T3, T6, T12, and T18.

Total Cohort (N = 1250)	T3, 1250 (100%)	T6, 1224 (97.9%)	T12, 181 (14.5%)	T18, 87 (7.0%)
Anosmia, n (%)	12 (1.0%)	7 (0.6%)	3 (1.7%)	2 (2.3%)
Dysgeusia, n (%)	12 (1.0%)	7 (0.6%)	2 (1.1%)	1 (1.1%)

**Table 3:** Anosmia at FUP T3.

	Anosmia: No (n = 1238)	Anosmia: Yes (n = 12)	*p*-Value
**Gender**			
Male, n (%)	591 (47.7%)	9 (75.0%)	0.081
Female, n (%)	647 (52.3%)	3 (25.0%)	
**Comorbidities, n (%)**			0.152
No	1097 (88.6%)	9 (75.0%)	
Yes	141 (11.4%)	3 (25.0%)	
**Severity, n (%)**			0.492
Asymptomatic	108 (8.7%)	0 (0.0%)	
Mild	1107 (89.4%)	12 (100%)	
Moderate	23 (1.9%)	0 (0.0%)	
**Hospitalization, n (%)**			0.224
No	1213 (98.0%)	11 (91.7%)	
Yes	25 (2.0%)	1 (8.3%)	
**COVID-19 variant, n (%)**			
Original	35 (2.8%)	2 (16.7%)	0.047
Alpha	72 (5.8%)	1 (8.3%)	0.516
Delta	240 (19.4%)	6 (50.0%)	0.017
Omicron	891 (72.0%)	3 (25.0%)	0.001
**Vaccination, n (%)**			0.714
No	993 (80.2%)	9 (75.0%)	
Yes	245 (19.8%)	3 (25.0%)	
**Complete vaccination, n (%)**			0.669
No	1068 (86.6%)	10 (83.3%)	
Yes	165 (13.4%)	2 (16.7%)	
**Acute anosmia**			
No	1183 (95.6%)	3 (25.0%)	0
Yes	55 (4.4%)	9 (75.0%)	
**Acute dysgeusia**			0
No	1193 (96.4%)	4 (33.3%)	
Yes	45 (3.6%)	8 (66.7%)	
**Resuscitation**			
No	1238 (100.0%)	12 (100.0%)	

**Table 4:** Dysgeusia at FUP T3.

	Dysgeusia: No (n = 1238)	Dysgeusia: Yes (n = 12)	*p*-Value
**Gender**			
Male, n (%)	593 (47.9%)	7 (58.3%)	0.567
Female, n (%)	645 (52.1%)	5 (41.7%)	
**Comorbidities, n (%)**			0.152
No	1097 (88.6%)	9 (75.0%)	
Yes	141 (11.4%)	3 (25.0%)	
**Severity, n (%)**			0.492
Asymptomatic	108 (8.7%)	0 (0.0%)	
Mild	1107 (89.4%)	12 (100%)	
Moderate	23 (1.9%)	0 (0.0%)	
**Hospitalization, n (%)**			1.00
No	1212 (97.9%)	12 (100%)	
Yes	26 (2.1%)	0 (0.0%)	
**COVID-19 variant, n (%)**			
Original	35 (2.8%)	2 (16.7%)	0.047
Alpha	71 (5.7%)	2 (16.7%)	0.152
Delta	241 (19.5%)	5 (41.7%)	0.067
Omicron	891 (72.0%)	3 (25.0%)	0.001
**Vaccination, n (%)**			0.669
No	993 (80.2%)	9 (75.0%)	
Yes	245 (19.8%)	3 (25.0%)	
**Complete vaccination, n (%)**			0.714
No	993 (80.2%)	9 (75.0%)	
Yes	245 (19.8%)	3 (25.0%)	
**Acute anosmia**			
No	1184 (95.6%)	2 (16.7%)	0
Yes	54 (4.4%)	10 (83.3%)	
**Acute dysgeusia**			0
No	1193 (96.4%)	4 (33.3%)	
Yes	45 (3.6%)	8 (66.7%)	
**Resuscitation**			
No	1238 (100.0%)	12 (100.0%)	

**Table 5:** Anosmia at FUP T6.

	Anosmia: No (n = 1217)	Anosmia: Yes (n = 7)	*p*-Value
**Gender**			
Male, n (%)	575 (47.2%)	5 (71.4%)	0.266
Female, n (%)	642 (52.8%)	2 (28.6%)	
**Comorbidities, n (%)**			0.571
No	1079 (88.6%)	6 (85.7%)	
Yes	138 (11.3%)	1 (14.3%)	
**Severity, n (%)**			0.656
Asymptomatic	108 (8.9%)	0 (0.0%)	
Mild	1086 (89.2%)	7 (100%)	
Moderate	23 (1.9%)	0 (0.0%)	
**Hospitalization, n (%)**			1.00
No	1191 (97.9%)	7 (100.0%)	
Yes	26 (2.1%)	0 (0.0%)	
**COVID-19 variant, n (%)**			
Original	35 (2.9%)	1 (14.3%)	0.189
Alpha	75 (5.9%)	1 (14.3%)	0.35
Delta	241 (19.8%)	5 (71.4%)	0.005
Omicron	869 (21.4%)	0 (0.0%)	0
**Vaccination, n (%)**			
No	976 (80.2%)	6 (85.7%)	
Yes	241 (19.8%)	1 (14.3%)	1
**Complete vaccination, n (%)**			
No	1051 (86.7%)	7 (100.0%)	
Yes	161 (13.3%)	0 (0.0%)	0.604
**Acute anosmia**			
No	1161 (95.4%)	1 (14.3%)	0
Yes	56 (4.6%)	6 (85.7%)	
**Acute dysgeusia**			
No	1171 (96.2%)	2 (28.6%)	0
Yes	46 (3.8%)	5 (71.4%)	
**Resuscitation**			
No	1217 (100.0%)	7 (100.0%)	

**Table 6:** Dysgeusia at FUP T6.

	Dysgeusia: No (n = 1217)	Dysgeusia: Yes (n = 7)	*p*-Value
**Gender**			
Male, n (%)	576 (47.3%)	4 (57.1%)	0.714
Female, n (%)	641 (52.7%)	3 (42.9%)	
**Comorbidities, n (%)**			0.571
No	1079 (88.6%)	6 (85.7%)	
Yes	138 (11.3%)	1 (14.3%)	
**Severity, n (%)**			0.656
Asymptomatic	108 (8.9%)	0 (0.0%)	
Mild	1086 (89.2%)	7 (100%)	
Moderate	23 (1.9%)	0 (0.0%)	
**Hospitalization, n (%)**			1.00
No	1191 (97.9%)	7 (100.0%)	
Yes	26 (2.1%)	0 (0.0%)	
**COVID-19 variant, n (%)**			
Original	35 (2.9%)	1 (14.3%)	0.189
Alpha	72 (5.9%)	1 (14.3%)	0.35
Delta	241 (19.8%)	5 (71.4%)	0.005
Omicron	869 (21.4%)	0 (0.0%)	0
**Vaccination, n (%)**			
No	976 (80.2%)	6 (85.7%)	
Yes	241 (19.8%)	1 (14.3%)	1
**Complete vaccination, n (%)**			
No	1051 (86.7%)	7 (100.0%)	
Yes	161 (13.3%)	0 (0.0%)	0.604
**Acute anosmia**			
No	1161 (95.4%)	1 (14.3%)	0
Yes	56 (4.6%)	6 (85.7%)	
**Acute dysgeusia**			
No	1171 (96.2%)	2 (28.6%)	0
Yes	46 (3.8%)	5 (71.4%)	
**Resuscitation**			
No	1217 (100.0%)	7 (100.0%)	

**Table 7:** Anosmia at FUP 12.

	Anosmia: No (n = 178)	Anosmia: Yes (n = 3)	*p*-Value
**Gender**			
Male, n (%)	76 (42.7%)	0 (0.0%)	0.081
Female, n (%)	102 (57.3%)	3 (100.0%)	
**Comorbidities, n (%)**			0.324
No	157 (88.2%)	2 (66.7%)	
Yes	21 (11.8%)	1 (33.3%)	
**Severity, n (%)**			0.64
Asymptomatic	27 (15.2%)	0 (0.0%)	
Mild	137 (77.0%)	3 (100%)	
Moderate	14 (7.9%)	0 (0.0%)	
**Hospitalization, n (%)**			1.00
No	167 (93.8%)	3 (100.0%)	
Yes	11 (6.2%)	0 (0.0%)	
**COVID-19 variant, n (%)**			
Original	32 (18.0%)	1 (33.3%)	0.455
Alpha	69 (38.8%)	1 (33.3%)	1
Delta	70 (39.3%)	1 (33.3%)	1
Omicron	7 (3.9%)	0 (0.0%)	1
**Vaccination, n (%)**			0.097
No	173 (97.2%)	2 (66.7%)	
Yes	5 (2.8%)	1 (33.3%)	
**Complete vaccination, n (%)**			1
No	176 (98.9%)	3 (100.0%)	
Yes	2 (1.1%)	0 (0.0%)	
**Acute anosmia**			
No	155 (87.1%)	0 (0.0%)	0.003
Yes	23 (12.9%)	3 (100%)	
**Acute dysgeusia**			0.036
No	159 (89.3%)	1 (33.3%)	
Yes	19 (10.7%)	2 (66.7%)	
**Resuscitation**			
No	178 (100.0%)	3 (100.0%)	

**Table 8:** Dysgeusia at FUP 12.

	Dysgeusia: No (n = 179)	Dysgeusia: Yes (n = 2)	*p*-Value
**Gender**			
Male, n (%)	77 (43.0%)	0 (0.0%)	0.189
Female, n (%)	102 (57.0%)	2 (100.0%)	
**Comorbidities, n (%)**			1
No	157 (88.7%)	2 (100.0%)	
Yes	22 (12.3%)	0 (0.0%)	
**Severity, n (%)**			0.744
Asymptomatic	27 (15.2%)	0 (0.0%)	
Mild	138 (77.1%)	2 (100%)	
Moderate	14 (7.8%)	0 (0.0%)	
**Hospitalization, n (%)**			1.00
No	167 (93.8%)	2 (100.0%)	
Yes	11 (6.2%)	0 (0.0%)	
**COVID-19 variant, n (%)**			
Original	33 (18.4%)	0 (0.0%)	1
Alpha	69 (38.5%)	1 (50.0%)	1
Delta	70 (39.3%)	1 (50.0%)	1
Omicron	7 (3.9%)	0 (0.0%)	1
**Vaccination, n (%)**			0.065
No	174 (97.2%)	2 (100.0%)	
Yes	2 (1.1%)	0 (0.0%)	
**Complete vaccination, n (%)**			1
No	177 (98.9%)	2 (100.0%)	
Yes	2 (1.1%)	0 (0.0%)	
**Acute anosmia**			
No	155 (87.1%)	0 (0.0%)	0.029
Yes	23 (13.4%)	2 (100%)	
**Acute dysgeusia**			0.219
No	159 (88.8%)	1 (50.0%)	
Yes	20 (11.2%)	1 (50.0%)	
**Resuscitation**			
No	179 (100.0%)	2 (100.0%)	

**Table 9:** Anosmia at FUP 18.

	Anosmia: No (n = 85)	Anosmia: Yes (n = 2)	*p*-Value
**Gender**			
Male, n (%)	34 (40.0%)	2 (100.0%)	0.089
Female, n (%)	51 (60%)	0 (0.0%)	
**Comorbidities, n (%)**			0.278
No	73 (85.9%)	1 (50.0%)	
Yes	12 (14.1%)	1 (50.05%)	
**Severity, n (%)**			0.707
Asymptomatic	12 (15.3%)	0 (0.0%)	
Mild	63 (74.1%)	2 (100%)	
Moderate	9 (10.6%)	0 (0.0%)	
**Hospitalization, n (%)**			1.00
No	78 (91.8%)	2 (100.0%)	
Yes	7 (8.2%)	0 (0.0%)	
**COVID-19 variant, n (%)**			
Original	29 (34.1%)	1 (50.0%)	1
Alpha	49 (57.6%)	0 (0.0%)	0.188
Delta	7 (8.2%)	1 (50.0%)	0.178
Omicron	0 (0.0%)	0 (0.0%)	
**Vaccination, n (%)**			1
No	84 (98.8%)	2 (100.0%)	
Yes	1 (1.2%)	0 (0.0%)	
**Complete vaccination, n (%)**			
No	85 (100.0%)	2 (100.0%)	
Yes	0 (0.0%)	0 (0.0%)	
**Acute anosmia**			
No	72 (84.7%)	0 (0.0%)	0.028
Yes	13 (15.3%)	2 (100.0%)	
**Acute dysgeusia**			0.021
No	74 (87.1%)	0 (0.0%)	
Yes	11 (12.9%)	2 (100.0%)	
**Resuscitation**			
No	85 (100.0%)	2 (100.0%)	

**Table 10:** Dysgeusia at FUP 18.

	Dysgeusia: No (n = 86)	Dysgeusia: Yes (n = 1)	*p*-Value
**Gender**			
Male, n (%)	35 (40.7%)	1 (100.0%)	0.414
Female, n (%)	51 (59.3%)	0 (0.0%)	
**Comorbidities, n (%)**			1
No	73 (84.9%)	1 (100.0%)	
Yes	13 (15.1%)	0 (0.0%)	
**Severity, n (%)**			0.843
Asymptomatic	13 (15.1%)	0 (0.0%)	
Mild	64 (74.4%)	1 (100%)	
Moderate	9 (10.5%)	0 (0.0%)	
**Hospitalization, n (%)**			1.00
No	79 (91.9%)	1 (100.0%)	
Yes	7 (8.1%)	0 (0.0%)	
**COVID-19 variant, n (%)**			
Original	30 (34.9%)	0 (0.0%)	1
Alpha	49 (57.0%)	0 (0.0%)	0.473
Delta	7 (8.1%)	1 (100.0%)	0.092
Omicron	0 (0.0%)	0 (0.0%)	
**Vaccination, n (%)**			1
No	85 (98.8%)	1 (100.0%)	
Yes	1 (1.2%)	0 (0.0%)	
**Complete vaccination, n (%)**			
No	86 (100.0%)	1 (100.0%)	
Yes	0 (0.0%)	0 (0.0%)	
**Acute anosmia**			
No	72 (83.7%)	0 (0.0%)	0.028
Yes	13 (16.3%)	1 (100.0%)	
**Acute dysgeusia**			0.021
No	74 (86.0%)	0 (0.0%)	
Yes	12 (14.0%)	1 (100.0%)	
**Resuscitation**			
No	86 (100.0%)	1 (100.0%)	

## Data Availability

Data are available upon request to the corresponding author.
